# Detection of ricin activity and structure by using novel galactose-terminated magnetic bead extraction coupled with mass spectrometric detection

**DOI:** 10.1016/j.ab.2021.114364

**Published:** 2021-09-03

**Authors:** Kaitlin Hoyt, John R. Barr, Suzanne R. Kalb

**Affiliations:** Centers for Disease Control and Prevention, Atlanta, GA, USA

**Keywords:** Ricin, Mass spectrometry, MALDI, Biotyper, Galactose affinity

## Abstract

Ricin is a toxic protein derived from the castor bean plant (*Ricinus communis*) and has potential for bioterrorism or criminal use. Therefore, sensitive and rapid analytical methods are needed for its confirmatory detection in environmental samples. Our laboratory previously reported on the development of a confirmatory method to detect ricin involving antibody capture of ricin followed by mass spectrometric detection of ricin’s enzymatic activity and of tryptic fragments unique to ricin. Here, we describe a novel ricin capture method of magnetic beads coated with 4-aminophenyl-1-thiol-β-galactopyranoside, using ricin’s lectin characteristics. The assay has been adapted for use on a simple, benchtop MALDI-TOF MS mass spectrometer common in clinical microbiology laboratories. Validation of the novel assay includes establishment of a limit of detection, and an examination of assay selectivity. The limit of detection of the enzymatic activity method is 8 ng/mL and 500 ng/mL for the confirmatory tryptic fragment assay. The assay is highly selective with no cross-reactivity from near neighbors and highly specific with a panel of 19 cultivars all testing positive. Additionally, there were no interferences found during testing of a panel of white powders. This allows for a confirmatory detection method for ricin in laboratories lacking expensive, sophisticated mass spectrometers.

## 1. Introduction

Ricin is a protein produced by the *Ricinus communis* or castor bean and is one of the most potent and deadly toxins known. Ricin is comprised of two polypeptide chains, a catalytic A chain and a lectin B chain; the A-chain of ricin exhibits rRNA N-glycosidase activity, while the B-chain facilitates cellular uptake [1]. Ricin is a type 2 ribosome-inactivating protein toxin (RIP-II toxin). RIP-II toxins inhibit protein synthesis by depurinating A4324 (a specific adenosine in mammals) of 28S ribosomal RNA [2]. The LD_50_ for ricin is estimated to be 5–10 μg/kg through inhalation and 20 mg/kg oral ingestion in humans [3,4]. Approximately 1.8 million castor bean plants are grown globally in the production of castor oil and the extraction process to produce ricin is not difficult to accomplish [5]. Ricin is approximately 1–5% of the castor bean by weight and one crushed castor bean contains approximately 1–2 mg of ricin/g [6].

Due to the lethal nature of ricin and the possibility of it being used for bioterrorism or criminal purposes, sensitive and rapid analytical methods are needed for confirmatory detection of ricin in environmental samples, food, and white powders. The confirmatory method developed at the CDC is a workflow that combines antibody capture, an *in vitro* activity assay, and a structural assay. The antibody capture uses magnetic beads coated with anti-ricin monoclonal antibodies. The activity assay analyzes the depurination of a synthetic RNA substrate and the structural assay includes a tryptic digestion followed by detection and quantification of peptides unique to ricin [7,8]. These methods often use very sensitive Liquid Chromatography- Tandem Mass Spectrometry (LC-MS/MS) instruments to analyze samples, detecting as low as 0.1 ng/mL [9]. The high-resolution LC-MS/MS structural assay can detect as little as 0.2 ng/mL [7]. While the sensitivity of the LC-MS/MS method is excellent for low limits of detection, a more cost-effective, space-conscious, and time-efficient method is needed for consideration in other laboratories and to increase the rapidness of response.

Many state and local public health laboratories have commercially-available versions of a benchtop MALDI-TOF MS (matrix-assisted laser desorption ionization time-of-flight mass spectrometer) system for microorganism identification. MALDI-TOF MS is a useful tool for a high- throughput and prompt analysis of complex mixtures by sorting ions based on their mass-to-charge ratio. Such systems are also common in many academic centers and advanced hospital clinical microbiology laboratories. These MALDI-TOF MS instruments are often smaller or benchtop models and therefore, are crucial for smaller laboratories that don’t have accessible space for larger LC-MS/MS instruments. Additionally, LC-MS/MS instruments are typically complex and often require operators with a sophisticated skill set for operation. Lastly, MALDI-TOF MS provides a swift analysis, taking less than 15 s per spot, which would be essential for a large influx of samples. A confirmatory ricin detection method that can be done entirely on a benchtop MALDI-TOF MS system would greatly expand the capacity and decrease the time required to detect ricin in the event of a criminal or bioterrorism incident to protect public health.

Our previous publications used antibody coated magnetic beads to extract ricin from environmental, food, and white powders[7,8]. We desired to explore alternatives to the use of antibodies for ricin extraction. One promising option involved production of galactose-coated micronanoparticles; however, the procedure for bead manufacture appeared complicated and lengthy, involving a procedure of nearly 1000 words, 26 separate chemicals (some of which are quite dangerous), and 6–7 days to complete, involving multiple steps of column chromatography[10]. More recently, several publications have detailed success with ricin extraction using functionalized gold nanoclusters[11] gold nanoparticles[12], and lactose-agarose beads[13]. Many of these techniques demonstrated the ability to capture ricin rather selectively from a variety of matrices including serum, a rather complex matrix.

Additionally, Sten-Ake Fredriksson recently reported the use of a galactosyl-sepharose affinity column to extract ricin toxin prior to enzymatic digestion and LC-MS/MS analysis [14]. This method worked well with a low detection limit of 10 ƒmol/mL (0.65 ng/mL), however, the affinity columns require a chromatographic system for their use, limiting the sample volume for analysis. To allow both the activity and structural assays to be performed on a benchtop MALDI TOF MS, we explored the use of 4-aminophenyl-1-thiol-β-galactopyranoside sugar (4-APTG) to coat BcMag™ amine-terminated magnetic beads. The use of magnetic beads rather than an affinity column allows for these environmental samples to be analyzed for ricin activity and structure on the MALDI-TOF MS in the absence of chromatography equipment.

## 2. Materials and methods

### 2.1. Safety considerations

Ricin is toxic and requires appropriate safety measures. The following protocol was designed to conjugate amine-containing ligands and to conduct an activity assay and tryptic digest. All toxin was handled in select agent registered space in a level 2 biosafety cabinet. Appropriate safety control measures (including engineering, administrative, and personal protective equipment) were used for all procedures based on a site-specific risk assessment that identified physical, health, and procedural hazards.

### 2.2. Chemicals and reagents

Ricin and its separate chains were obtained from Vector Laboratories (Burlingame, CA). 5 μm BcMag™ Amine-Terminated Magnetic Beads (Catalog number: FA-104) were obtained from Bioclone Inc. 4-aminophenyl-1-thiol-β-D-galactopyranoside (Catalog Number: 4014175) was obtained from Bachem. The substrate, RNA14A, (rCrGrCrGrCrGArGrArGrCrGrCrG) was synthesized by Integrated DNA Technologies (IDT). Both the oligonucleotide calibration standard (Catalog Number: 206200) and the MBT Biotarget 96 disposable plate (Catalog Number: 1840375) were purchased from Bruker Daltonics Inc. Our *communis cultivars* [15] and our near neighbors [16] were prepared according to the exact specifications stated previously. Instant dry milk was purchased from a local grocery store. White powder stock solutions were prepared at 1 mg/mL in water. All chemicals were obtained from Sigma-Aldrich unless otherwise stated.

### 2.3. Buffer preparation

Buffers, including the coupling buffer, 5% Glutaraldehyde solution, reaction stop buffer, and bead storage buffer were prepared according to the manufacturer’s instructions with the following exceptions: the 5% Glutaraldehyde solution used 70% Glutaraldehyde rather than 25%, and the bead storage buffer used was PBST (Phosphate buffered saline pH 7.4 with TWEEN-20) which was made by adding deionized water to lyophilized PBST to obtain a final solution of 0.1 M.

### 2.4. Bead activation

Beads were activated according to the manufacturer’s instructions using 1 mL of beads.

### 2.5. Coupling of sugar

**Fig. 1.**
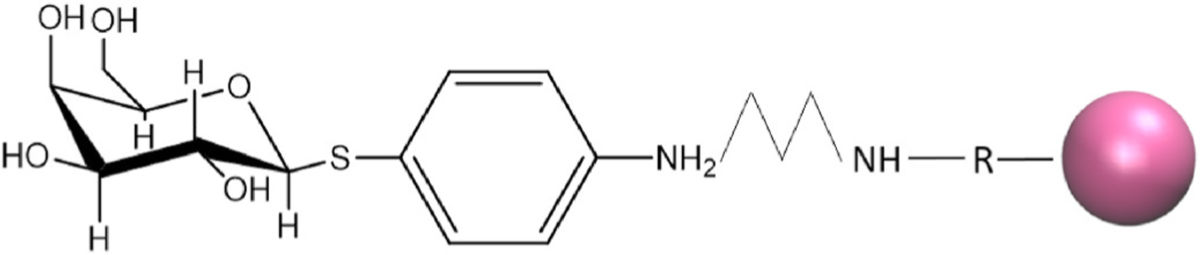
5 μm BcMag™ Amine-Terminated Magnetic Beads covalently bound to 4-aminophenyl-1-thiol-β-D-galactopyranoside. The primary amine on the 4-aminophenyl-1-thiol-β-D-galactopyranoside couples with the primary amine functioning group on the surface of the silica-based supermagnetic bead [17].

The amine on the surface of the BcMag™ amine-terminated magnetic beads (Bioclone Inc.) was covalently conjugated to the primary amine present on the 4-APTG structure [17] (Fig. 1). A 2 mg/mL solution of 4-aminophenyl-1-thiol-β-D-galactopyranoside (4-APTG) was prepared in coupling buffer. The 2 mg/mL solution of 4-APTG was added to the previously activated 5 μm BcMag™ Amine-Terminated Magnetic Beads and vortexed to mix. The activated 5 μm BcMag™ Amine-Terminated Magnetic Beads and 2 mg/mL 4-APTG solution were placed on gentle rotation on a laboratory rotisserie/laboratory tube rotator at 30 rpm for 12 h or overnight. After incubation was completed the Falcon tube was placed on the separator magnet and the supernatant was removed. A reaction stop buffer (4 mL for 1 mL of beads) was then added to the beads and placed on gentle rotation on a laboratory rotisserie/laboratory tube rotator at 30 rpm for 30 min. After the incubation was completed the Falcon tube was placed on the separator magnet and the supernatant was removed. The 4-APTG -Terminated Magnetic Beads were washed in PBST (1 mL of PBST per 1 mL of beads) and vortexed at 1500 rpm for 30 s. The previously mentioned process was repeated twice more. The 4-APTG -Terminated Magnetic Beads were suspended in PBST (1 mL per 1 mL of beads) and stored at 4°C for up to 6 months.

### 2.6. Antibody bead preparation

Antibody-coated magnetic beads were prepared as previously described [15].

### 2.7. Ricin activity reaction & MALDI-TOF MS detection

The ricin toxin, ranging between 1 μg/mL and 1 ng/mL, was spiked into 0.5 mL of phosphate buffered saline with tween-20 (PBST), mixed with an aliquot of 40 μL of 4-APTG -terminated beads, and the solution was kept at constant agitation using a KingFisher Flex instrument (ThermoFisher Scientific) for 1 h at room temperature and then washed for 1 min with a PBST wash. Following the capture of the toxin, the beads were reconstituted in a mixture of reaction buffer and the RNA14A. The RNA14A was dissolved in deionized water to a final concentration of 1 mM. The reaction buffer consisted of 5 mM ammonium citrate with 1 mM ethyl-enediaminetetraacetic acid (EDTA) adjusted to a pH of 4.1. Reaction solutions contained toxin bound 4-APTG-terminated beads in 18 μL of reaction buffer and 2 μL of the RNA14A substrate solution that were then incubated at 45°C for 4 h. After the incubation, 2 μL of supernatant from the reaction were mixed with 18 μL of MALDI matrix solution. The MALDI matrix consisted of 735 mM 3-hydroxypicolinic acid, 40 mM ammonium citrate, and 0.1% trifluoroacetic acid in 50/50 acetonitrile/deionized water. A 2 μL aliquot of the supernatant and MALDI matrix were spotted onto the MBT Biotarget 96 disposable plate. The spots were dried in the biological safety cabinet. This process was repeated once more on the same spots. The samples were placed in a 4°C refrigerator for storage. The mass spectra were collected for each spot by analyzing 200 laser shots from 1000 to 6000 *m/z* in positive ion linear mode with internal calibration on the Bruker Microflex System (Bruker Daltonics Inc.). The spectra were processed by using a Mass Control List in the Bruker FlexAnalysis software that identifies the masses of the intact RNA14A substrate, 4522 *m/z*, and the depurinated product, 4404 *m/z*, with y-axis corresponding to intensity as defined by peak height with autoscaling.

For samples with added lactase, 500 μL of a 2 mg/mL lactase solution in water was added directly to 500 μL of a 1 mg/mL white powder sample solution in 1X PBST. This mixture of lactase solution and white powder sample solution were incubated at 27°C for 30 min with constant rotation. The remainder of the protocol is as above. Cultivar extracts were prepared as published previously [15] and diluted in 1X PBST prior to analysis.

### 2.8. Tryptic digestion & MALDI-TOF MS detection

The supernatant (confirmed to be absent of active ricin) was removed from the beads and the beads were reconstituted in 18 μL of 50 mM ammonium bicarbonate and 2 μL of Promega gold trypsin at 0.5 mg/mL. The beads and solution were incubated at 52°C for 20 min for the tryptic digest and 95°C for 10 min for safety purposes. After the completion of the incubation, the 20 μL of supernatant was removed from the beads. The 20 μL of supernatant was concentrated and purified utilizing a C18 ziptip (Millipore) and 2 μL of CHCA matrix was eluted off the ziptip and directly onto a MBT Biotarget 96 disposable plate to analyze. The CHCA matrix was comprised of 10 mg of CHCA vial (Sigma Aldrich C8982), 1 M ammonium citrate, and 0.1% trifluoroacetic acid in a 50/50 acetonitrile/water mixture. The mass spectra were collected for each spot by analyzing from 1000 to 6000 *m/z* in positive ion linear mode on the Bruker Microflex System (Bruker Daltonics Inc.). The spectra were processed by using a Mass Control List in the Bruker FlexAnalysis software that identifies the masses of six unique tryptic fragments, 897 *m/z*, 1046 *m/z*, 2307 *m/z*, 2994 *m/z,* 3283 *m/z,* or 3309 *m/z*, to confirm the sample is ricin.

## 3. Results and discussion

### 3.1. Optimizing toxin recovery by utilizing galactose-terminated beads

Antibody-coated beads have been used successfully to recover and bind ricin toxin in several activity assays [7,8]. However, there are disadvantages to antibody-coated beads, such as lot-to-lot variability of antibodies and the need to store antibodies under stringent conditions. Interested in an alternative extraction approach, we utilized published information by Sten-Ake Fredriksson to explore the possibility of using a sugar to bind ricin [14]. The amine on the surface of the BcMag™ amine-terminated magnetic beads (Bioclone Inc.) could be covalently conjugated to the primary amine present on the 4-APTG structure [17] (Fig. 1). These beads, while significantly different from the antibody-coated beads, also function as affinity capture beads and use similar protocols as the antibody-coated beads, with only slight changes for optimal toxin capture. Table 1 indicates the differences in protocol to make and use the beads, as well as, the difference in price per reaction for the beads. The price for beads per reaction was calculated based on the cost of reagents, amount of beads used during a reaction, the sugar/antibody, and the magnetic beads themselves. The cost could be beneficial for laboratories, especially for those with limited resources and funding since the galactose-terminated beads are nearly half the cost of the antibody-coated beads.

### 3.2. Comparison of antibody-coated beads and galactose-terminated beads

**Fig. 2.**
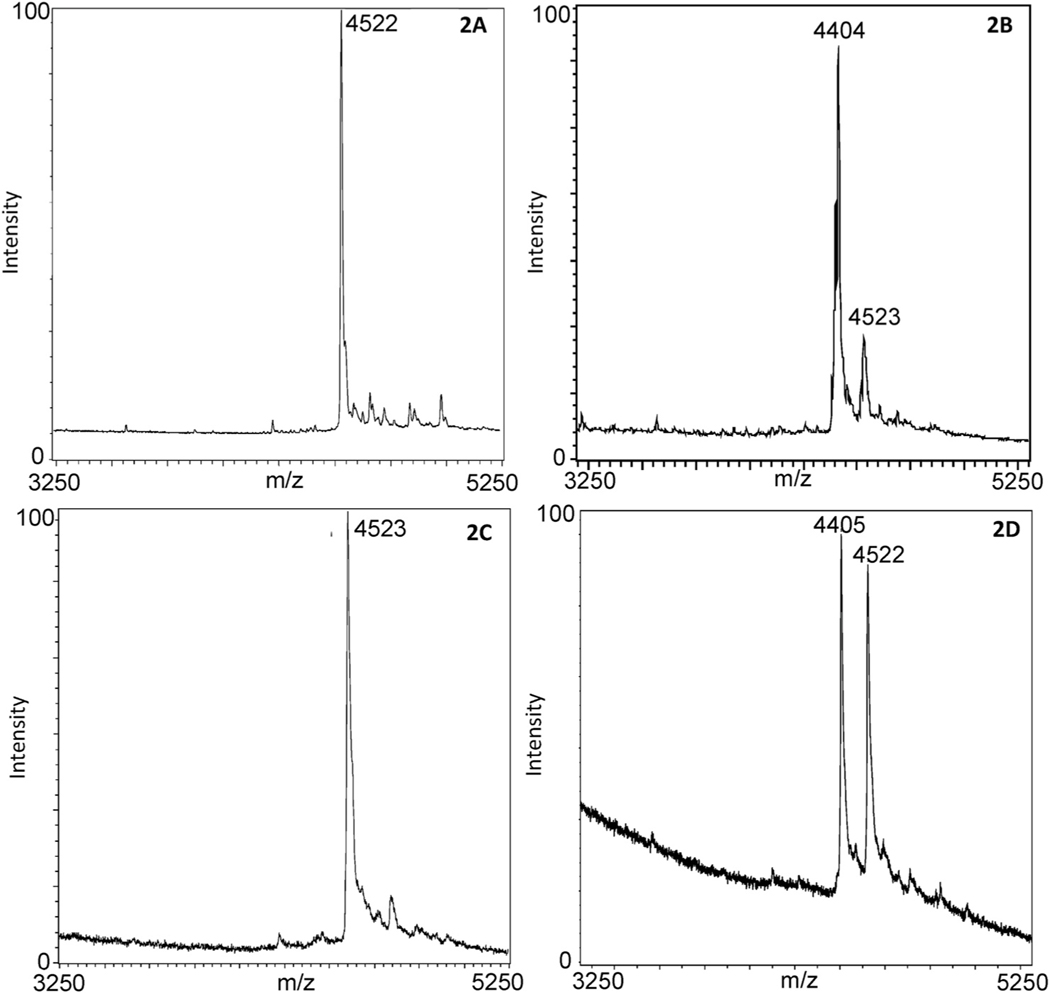
The comparison between antibody-coated beads and galactose-terminated beads for the activity portion of the assay. (A) Mass spectrum of 14-A substrate, present at 4522 *m/z*, containing no ricin analyzed using antibody-coated beads. (B) Mass spectrum of a sample containing 500 ng/mL of ricin with depurinated peak at 4404 *m/z*, and intact 14-A substrate at 4523 *m/z*, analyzed using antibody-coated beads. (C) Mass spectrum of 14-A substrate, present at 4523 *m/z*, containing no ricin analyzed using galactose-terminated beads. (D) Mass spectrum of a sample containing 500 ng/mL of ricin with depurinated peak at 4405 *m/z*, and intact 14-A substrate at 4522 *m/z*, analyzed using galactose-terminated beads.

Fig. 2A and B represent the extraction of ricin through antibody-coated beads, one sample in the absence of ricin (2A) and one sample in the presence of ricin (2B). Fig. 2C and D represent the extraction of ricin through the galactose-terminated beads, in the absence of ricin (2C) and in the presence of ricin (2D), respectively. Unreacted RNA14A substrate indicating the absence of enzymatically active ricin is present at 4522–4523 *m/z* and depurinated RNA14A substrate showing the presence of enzymatically active ricin is present at 4404–4405 *m/z*. Both the antibody-coated beads and galactose-terminated bead mass spectra illustrate depurination of the RNA14A substrate by the captured ricin in the activity portion of the assay (Figures 2B and D). More of the depurination product is observed in the antibody-captured ricin experiment. This could be because the antibody-beads are more effective in extracting ricin or that the antibody-bound ricin is more enzymatically active. Nonetheless, both the antibody-coated beads and the galactose-terminated beads can be used to extract ricin, and the extracted ricin remains enzymatically active for analysis in both cases.

However, detection of the enzymatic activity of ricin alone does not confirm the presence of ricin in a sample as ricin has the same enzymatic activity of other RIP-II toxins. To confirm ricin, we performed a peptide mapping approach to distinguish ricin from other RIP-II toxins due to the amino acid composition of signature ricin peptides. Together, this workflow yields confirmatory detection for the presence of ricin in a sample. The second portion of the workflow, the peptide assay, involves tryptic digestion of the toxin followed by MALDI TOF mass spectrometric analysis of the tryptic fragments. The detection of masses that correspond to specific tryptic peptides found in ricin coupled with a positive result in the enzymatic activity assay is considered a positive test result for ricin while a positive result in just one of the two assays is considered as a presumptive positive result.

**Fig. 3.**
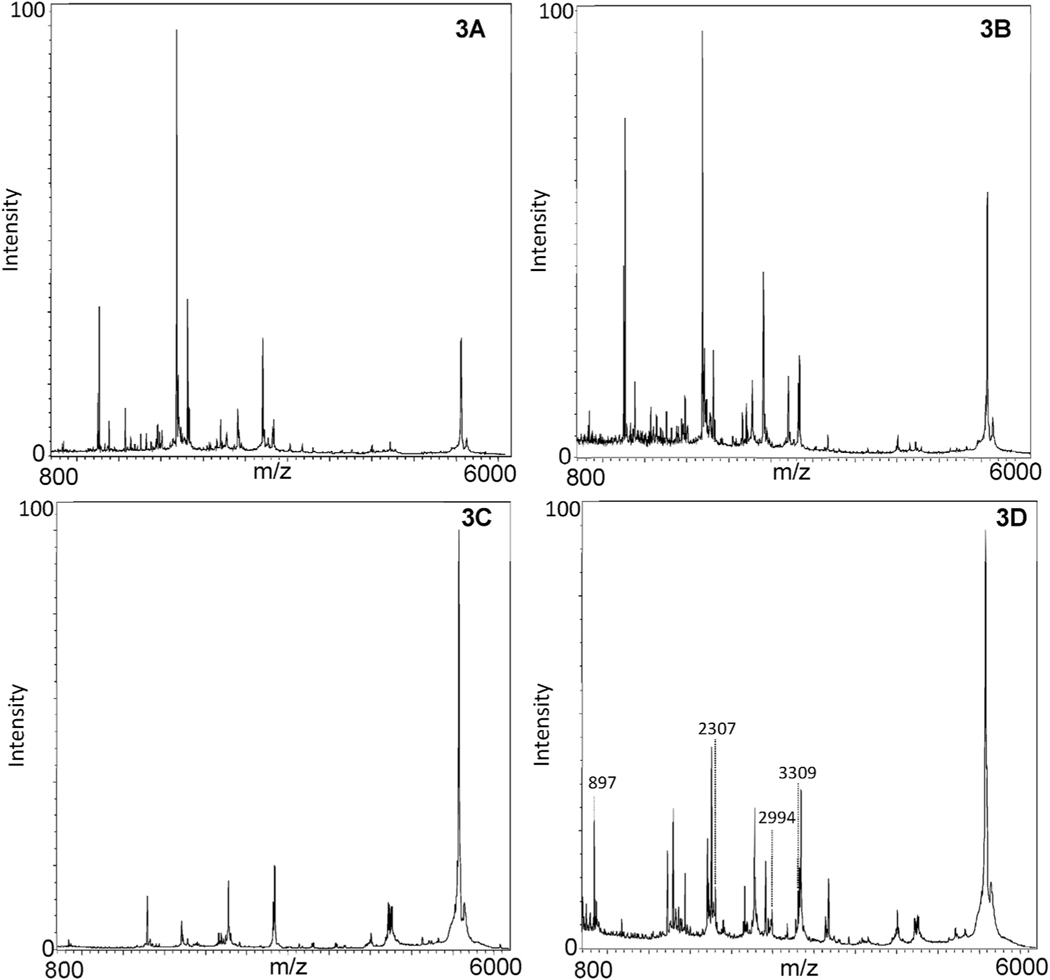
The comparison between antibody-coated beads and galactose-terminated beads for the structure portion of the assay. (A) Mass spectrum of tryptic digest of antibody-coated beads containing no ricin. (B) Mass spectrum of tryptic digest of antibody-coated beads containing 500 ng/mL of ricin. (C) Mass spectrum of tryptic digest of galactose-terminated beads containing no ricin. (D) Mass spectrum of tryptic digest of galactose-terminated beads containing 500 ng/mL of ricin. Ricin-specific tryptic fragments are only visible with the galactose-terminated beads, and are labeled in Fig. 3D.

Comparison of the performance of the antibody-coated beads and the galactose-terminated beads yields significant differences in the structure part of the assay. Fig. 3 represents the mass spectra generated following tryptic digestion of beads following exposure to blank samples (Fig. 3A and C) as well as samples containing ricin (Fig. 3B and D). No peaks specific for ricin are visible in the tryptic digest of the antibody-coated beads with ricin, as seen in the comparison of Fig. 3A (no ricin) and 3B (ricin). However, the galactose-terminated beads performed significantly better in the peptide mapping assay than the antibody coated beads. Fig. 3C is the mass spectrum generated by the tryptic digest of galactose-terminated beads from a sample absent of ricin. Fig. 3D is the mass spectrum from a tryptic digest of galactose-terminated beads bound ricin. The most significant difference between Fig. 3B and D is that Figure 3D has four visible tryptic fragments at 897 *m/z*, 2307 *m/z*, 2994 *m/z* and 3309 *m/z*, which correspond to tryptic fragments specific to ricin that are not found in other proteins including RCA120 (Table 2). These peaks are absent in the tryptic digest of antibody-coated beads exposed to the same level of ricin, and these peaks are also not present in tryptic digests of galactose-terminated beads absent of ricin.

Although the antibody-coated beads extract ricin as seen in the depurination of RNA14A in the activity assay, the antibody-coated beads do not detect ricin specific peptide peaks after the tryptic digestion of the ricin-bound antibody beads. We doubled the amount of antibody-coated beads used for extraction of ricin to determine if the lack of detection of ricin specific peptide peaks could be due to a lack of capacity of the antibody beads but it did not help in ricin detection (data not shown). This suggests that the lack of detection of ricin-specific peptides is due to the presence of interferences in the MALDI-TOF spectra from antibody digestion. It is also possible that the antibody-coated beads restrict tryptic digestion of ricin such that the tryptic fragments are not produced at a level needed for detection on a simple instrument. To obtain a positive result for ricin in this assay, both the activity assay and the peptide mapping assay must be positive and peptide mass must be consistent with ricin and not shared by the negative control or other similar ribosome-inactivating proteins.

### 3.3. Activity and structural limit of detection comparison with antibody-coated beads and galactose -terminated beads

Depurination of the RNA14A substrate is visible in mass spectra for both antibody-coated beads and galactose-terminated beads as seen in Fig. 2B and D. Table 3 compares the lowest limit of detection (LOD) possible with antibody-coated beads at 50 pg/mL, while the galactose-terminated beads show that their LOD is at 8 ng/mL after twenty replicates of the activity assay. The structural LOD for the galactose- terminated beads is 500 ng/mL, while the LOD for antibody beads was not detectable at the highest level tested (1 μg/mL), which was confirmed after twenty analytical analysis with 95% confidence. There is more than a 10-fold difference between the two LODs and the galactose-terminated beads lack the sensitivity of the antibody-coated beads for the detection of the enzymatic activity of ricin. However, the lower LOD for the activity assay afforded by the antibody-coated beads is offset by the inability of the antibody-coated beads to be used to detect ricin tryptic fragments in this assay. With the possibility of depurination of the RNA14A substrate by other selected RIPs, a positive result for ricin requires that both the activity and peptide mapping portions of the workflow must be positive.

### 3.4. White powder panel

Ricin’s appearance, in solid form, is a white crystalline powder. This white powder can often be confused with other readily available white powders. Additionally, ricin can be mixed with other materials. Unknown white powders are often received for environmental sample testing and can be perceived as a biological threat. A panel of 16 white powders, listed in Tables 4 and 5, were tested with and without 1 μg/mL of ricin and the white powders themselves were tested at 1 mg/mL solutions, except flour and baking powder, which were tested at 0.5 mg/mL due to bead clumping. This experiment evaluated if the negative control white powders would test falsely positive for ricin or if the white powders tested with spiked ricin would interfere or inhibit detection of the ricin. The results of this experiment are in Table 4 (activity) and Table 5 (structure). The white powders absent of ricin all correctly identified no ricin in the activity assay (Table 4). In the presence of ricin, all tested powders correctly identified ricin in the samples, with the exception of parmesan cheese and instant dry milk.

When examining the presence or absence of ricin biomarkers following a tryptic digest as confirmation, all tested powders correctly identified ricin as not being present (Table 5). Lastly, most tested powders in the ricin structure correctly identified ricin as being present when ricin was spiked into the white powders at the 1 μg/mL level by identifying at least two of the six biomarkers at 897 *m/z*, 1046 *m/z*, 2307 *m/z*, 2994 *m/z,* 3283 *m/z,* or 3309 *m/z*. The exceptions here are once again the parmesan cheese and instant dry milk samples. Although this is not a quantitative assay, it is true that the depurinated peak grows more intense in the presence of increased active ricin. Additionally, the tryptic digest fragments unique to ricin grow more intense in the presence of increased ricin. Based on the intensity of the depurinated RNA14A substrate and tryptic fragments, it appears that the majority of the white powder panel did not inhibit the activity of the assay or the peptide mapping component of the assay.

### 3.5. Assay improvement with addition of lactase

Instant dry milk and parmesan cheese were tested during the white powders panel and did not produce the expected results, as the ability of ricin detection was inhibited in both assays. It is possible that instant dry milk and grated parmesan cheese would be received as an environmental sample and therefore, we explored an alternate strategy to mitigate these matrices which both contain lactose. We found it important to address the instant dry milk and parmesan cheese testing more extensively and wanted to eliminate or minimize the interference in this assay.

Milk’s major carbohydrate is lactose, which varies in concentration from 0 to 10%, w/w [18]. Lactose is a disaccharide composed of the monosaccharides D-glucose and D-galactose, joined in a β−1,4-glycosidic linkage [19]. In a manuscript published by Christian Zentz, it was reported that every two molecules of lactose bind to one molecule of ricin, while one ricin protein binds to one galactose [20]. Therefore, lactose could interfere with ricin binding to galactose-terminated beads.

**Fig. 4.**
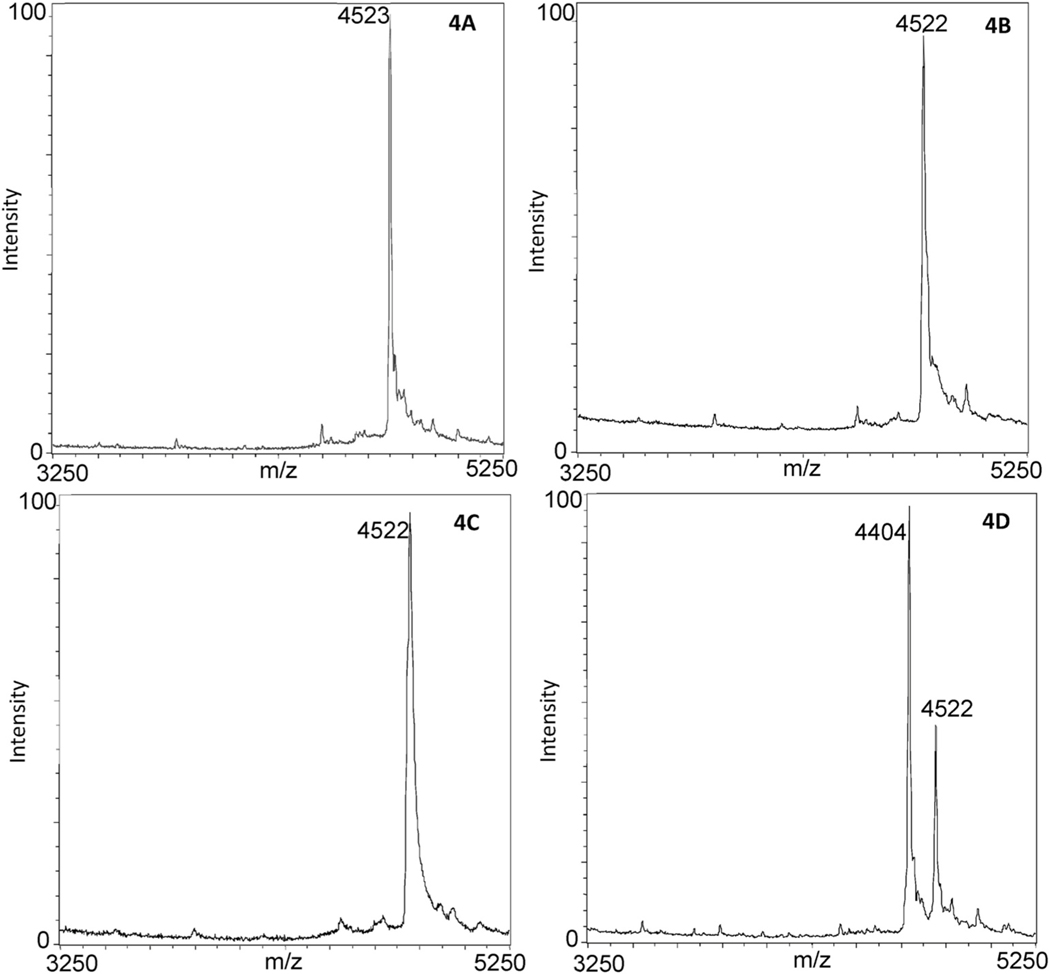
The comparison between instant dry milk with and without lactase. (A) Mass spectrum of 14-A substrate containing no ricin analyzed using galactose-bound beads and no 2 mg/mL lactase solution. (B) Mass spectrum of a sample containing 500 ng/mL of ricin and 14-A substrate analyzed using galactose-bound beads and no 2 mg/mL lactase. (C) Mass spectrum of 14-A substrate containing no ricin analyzed using galactose-bound beads and 2 mg/mL lactase. (D) Mass spectrum of a sample containing 500 ng/mL of ricin and 14-A substrate analyzed using galactose-bound beads and 2 mg/mL lactase solution.

Fig. 4A and B are mass spectra of instant dry milk in the absence (Fig. 4A) and presence (Fig. 4B) of ricin. Although the instant dry milk negative control yielded the correct result, it was discovered that no depurination of the RNA14A substrate occurred when 500 ng of ricin was introduced to the assay, yielding an increased LOD in the presence of instant dry milk. Therefore, it is plausible that the ricin is not binding to the galactose amine-terminated beads and possibly binding to the lactose, causing interference in the assay. To test this theory, lactase (β-galactosidase) was introduced to the assay to hydrolyze the instant dry milk lactose into D-glucose and D-galactose [21]. The lactase (2 mg/mL) was introduced to the 500 ng/mL ricin-spiked instant dry milk solutions and heated to 27°C for 30 min prior to the assay. Fig. 4C and D are mass spectra of the lactase-treated milk in the absence (Fig. 4C) and presence (Fig. 4D) of ricin. Depurination of the RNA14A substrate is absent in the negative control and quite apparent in the presence of ricin. The addition of lactase also allowed for detection of ricin spiked into parmesan cheese. This method therefore allowed for the binding of ricin to the galactose-bound bead and a depurination of the RNA14A substrate. This step was repeated with all white powders and did not affect the depurination of any ricin-spiked white powder and did not affect the negative controls, and therefore, was added to the beginning of the protocol to test unknown white powders.

### 3.6. Cultivars panel

*R. Communis* cultivars were obtained from several different countries to compare the detection of ricin present for the assay. Seeds from 18 *R. Communis* cultivars were obtained from J. Bradley Morris at the Plant Genetic Resources Conservation Unit, Agricultural Research Service (ARS), U.S. Department of Agriculture (USDA), in Griffin, Georgia [15]. As seen in Table 6, all cultivars tested were positive for ricin based upon their enzymatic activity and a tryptic digest of the cultivars after extraction.

### 3.7. Near-neighbors panel

As mentioned previously in the discussion, there are several other ribosome-inactivating proteins (RIPs) both Type I and Type II, referred to as near-neighbors, which could be mistaken with ricin during activity testing by depurination of the 14-A synthetic substrate. A panel was prepared of 12 near-neighbors to ricin consisting of crude extracts of related plants and purified portions of ricin and tested according to a prior protocol. *Ricinus communis*, RCA120,RCA120, and ricin B chain of the near neighbors presented as positive in the activity assay (Table 7).

An extract of *Ricinus communis* is expected to contain ricin, so this plant extract should test positive in the assay. The B-chain of ricin was purified from intact ricin, and it has been previously shown to contain a small amount of intact ricin [15]. Interestingly, the A-chain of ricin did not test positive in the activity assay, suggesting that the A-chain of ricin was not extracted by the galactose-coated beads. On examination of the structural assay for these near neighbors, the only positive upon testing in the structural assay (Table 7) was the control material, *Ricinus communis*. This work demonstrates the importance of the structural part of the assay which is necessary to confirm the presence of ricin.

## 4. Conclusion

The qualitative assay utilizing MALDI-TOF MS to detect the activity and structure of ricin was improved through the use of novel galactose-terminated beads. This assay was optimized by employing galactose-bound beads in place of antibody-coated beads to create a confirmatory assay, which would be necessary to differentiate between ricin and other ribosomal-inactivating proteins. The white powders, near-neighbors, and cultivars panels were all instrumental in determining the selectivity of the assay. Although this assay has limitations, they are outweighed by the potential of a confirmatory assay afforded by the use of galactose-terminated beads, which are not subject to lot-to-lot variation like antibodies are. This method would prove as a useful tool for quick, high-throughput detection of environmental ricin samples, and it could be used to confirm a preliminary positive result from a screening assay. In the future, this method could also be used to differentiate between proteins by identifying unique masses for each RIP in the structural portion of the assay as galactose has been reported to extract multiple RIP-II toxins [14].

## Figures and Tables

**Table 1:** Method parameter comparison between galactose-terminated beads and ricin antibody-coated beads, as estimated in 2020.

Method Element	Galactose-terminated Beads	Antibody-coated Beads
*Time to Make Beads*	*18 h*	*15 h*
*Relative Cost of Beads per Sample*	*$2.37*	*$4.04*
*Binding on KingFisher instrument*	*Yes*	*No*
*Binding on Plate Shaker*	*No*	*Yes*
*Amount of Beads*	40 μL	20 μL

**Table 2:** Tryptic fragments unique to ricin’s amino acid sequence observed during limit of detection experiments. Residues unique to ricin are underlined.

AA Sequence	Start	End	*m/z*
(R)VGLPINQR(F)	*84*	*91*	897
(R)GRLTTGADVR(H)	65	74	1046
*(*R)YTFAFGGNYDRLEQLAGNLR(E)	*150*	*169*	2307
*(*R)SFIICIQMISEAARFQYIEGEMRTR(I)	*202*	*226*	2994
(V)QSYTNFIRAVRGRLTTGADVRHEIPVLPNR(V)	*54*	*82*	3283
*(*R)AGNSAYFFHPDNQEDAEAITHLFTDVQNR(Y)	*121*	*149*	3309

**Table 3:** Activity Assay Limit of Detection Comparison with Antibody-coated Beads and Galactose Amine-terminated Beads after twenty replicates.

Limit of Detection	Galactose Amine-terminated Beads	Ricin Antibody-coated Beads
1 μg	Positive	Positive
100 ng	Positive	Positive
20 ng	Positive	Positive
10 ng	Positive	Positive
8 ng	Positive	Positive
5 ng	Negative	Positive
1 ng	Negative	Positive
50 pg	Negative	Positive
20 pg	Negative	Negative

**Table 4:** Presence of depurinated peak in the activity assay portion of the assay.

White Powder	Negative Control: Depurintated Product, 4404 m/z	Positive Control: Depurintated product, 4404 m/z
1X PBST (Control)	Negative	Positive
Miralax	Negative	Positive
Corn Starch	Negative	Positive
Parmesan cheese	Negative	Negative
Parmesan cheese with lactase	Negative	Positive
Powdered Sugar	Negative	Positive
Boric Acid	Negative	Positive
Glutamic Acid	Negative	Positive
Flour	Negative	Positive
Instant Dry Milk	Negative	Negative
Instant Dry Milk with lactase	Negative	Positive
Baking Powder	Negative	Positive
Baking Soda	Negative	Positive
Epsom Salt	Negative	Positive
Powdered Coffee Creamer	Negative	Positive
Chalk dust	Negative	Positive
Baby powder	Negative	Positive
Dish washing detergent	Negative	Positive
Crushed Tums	Negative	Positive

**Table 5:** Presence of at least 2 unique tryptic fragments to ricin’s amino acid sequence.

White Powder	Negative Control	Positive Control
1X PBST (Control)	Negative	Positive
Miralax	Negative	Positive
Corn Starch	Negative	Positive
Parmesan cheese	Negative	Negative
Parmesan cheese with lactase	Negative	Positive
Powdered Sugar	Negative	Positive
Boric Acid	Negative	Positive
Glutamic Acid	Negative	Positive
Flour	Negative	Positive
Instant Dry Milk	Negative	Negative
Instant Dry milk with lactase	Negative	Positive
Baking Powder	Negative	Positive
Baking Soda	Negative	Positive
Epsom Salt	Negative	Positive
Powdered Coffee Creamer	Negative	Positive
Chalk dust	Negative	Positive
Baby powder	Negative	Positive
Dish washing detergent	Negative	Positive
Crushed Tums	Negative	Positive

**Table 6:** Results from activity assay and structural assay (presence of at least 2 tryptic fragments unique to ricin).

Cultivar	Positive Control: Depurintated product, 4404 m/z	Tryptic Fragment of ricin
Us Florida; PI265508	Positive	Positive
Iran; PI222265	Positive	Positive
Peru; PI215770	Positive	Positive
Mexico; PI65446	Positive	Positive
Morocco; PI253621	Positive	Positive
Brazil; PI202711	Positive	Positive
Cuba; PI208839	Positive	Positive
US Virgin Islands; PI209326	Positive	Positive
Afghanistan; PI212115	Positive	Positive
Pakistan; PI217539	Positive	Positive
Tukey; PI167342	Positive	Positive
China; PI436592	Positive	Positive
Former Soviet Union; PI257654	Positive	Positive
Argentina; PI219767	Positive	Positive
India; PI173947	Positive	Positive
Puerto Rico; PI209132	Positive	Positive
Hale: PI624000	Positive	Positive
Syria; PI181916	Positive	Positive

**Table 7:** Presence of depurinated peak in the activity assay portion of the assay; presence of at least 2 tryptic fragments unique to ricin.

Near-neighbor	Positive Control: Depurintated product, 4404 m/z	Tryptic Fragment of ricin
Ricinus Communis (Control)	Positive	Positive
Viscum Album	Negative	Negative
Sambucus Ebulus	Negative	Negative
Senna Occidentails	Negative	Negative
Cinnamomum Camphora	Negative	Negative
Trichosanthes Kiriiowii	Negative	Negative
Iris Hollandica	Negative	Negative
Abrus Precatorius	Negative	Negative
Sambucus Nigra	Negative	Negative
Ricin A Chain	Negative	Negative
Ricin B Chain^a^	Positive	Negative
RCA120	Positive	Negative
Shiga Toxin	Negative	Negative

a Contains small amount of intact ricin.

## References

[1] Simmons BM, Stahl PD, Russell JH, Mannose receptor-mediated uptake of ricin toxin and ricin A chain by macrophages. Multiple intracellular pathways for a chain translocation, J. Biol. Chem. 261 (1986) 7912–7920. 3711116

[2] Montanaro L, Sperti S, Mattioli A, Testoni G, Stirpe F, Inhibition by ricin of protein synthesis in vitro. Inhibition of the binding of elongation factor 2 and of adenosine diphosphate-ribosylated elongation factor 2 to ribosomes, Biochem. J. 146 (1975) 127–131. 167711 10.1042/bj1460127 PMC1165282

[3] Bradberry SM, Dickers KJ, Rice P, Griffiths GD, Vale JA, Ricin poisoning, Toxicol. Rev. 22 (2003) 65–70. 14579548 10.2165/00139709-200322010-00007

[4] Moshiri M, Hamid F, Etemad L, Ricin toxicity: clinical and molecular aspects, Rep Biochem Mol Biol 4 (2016) 60–65. 27536698 PMC4986263

[5] Food and Agriculture Organization of the United Nations, Crops. www.fao.org/faostat, 2020.

[6] Gupta RC, Handbook of Toxicology of Chemical Warfare Agents, Elsevier Science, 2009.

[7] Kalb SR, Schieltz DM, Becher F, Astot C, Fredriksson SA, Barr JR, Recommended mass spectrometry-based strategies to identify ricin-containing samples, Toxins (Basel) 7 (2015) 4881–4894. 26610568 10.3390/toxins7124854 PMC4690104

[8] Wang D, Baudys J, Barr JR, Kalb SR, Improved sensitivity for the qualitative and quantitative analysis of active ricin by MALDI-TOF mass spectrometry, Anal. Chem. 88 (2016) 6867–6872. 27264550 10.1021/acs.analchem.6b01486 PMC5665012

[9] Becher F, Duriez E, Volland H, Tabet JC, Ezan E, Detection of functional ricin by immunoaffinity and liquid chromatography-tandem mass spectrometry, Anal. Chem. 79 (2007) 659–665. 17222034 10.1021/ac061498b

[10] Liu HZ, Tang JJ, Ma XX, Guo L, Xie JW, Wang YX, Galactose-functionalized magnetic iron-oxide nanoparticles for enrichment and detection of ricin toxin, Anal. Sci. 27 (2011) 19–24. 21233555 10.2116/analsci.27.19

[11] Selvaprakash K, Chen YC, Detection of ricin by using gold nanoclusters functionalized with chicken egg white proteins as sensing probes, Biosens. Bioelectron. 92 (2017) 410–416. 27836610 10.1016/j.bios.2016.10.086

[12] Selvaprakash K, Chen YC, Functionalized gold nanoparticles as affinity nanoprobes for multiple lectins, Colloids Surf B Biointerfaces 162 (2018) 60–68. 29149729 10.1016/j.colsurfb.2017.11.022

[13] Feldberg L, Elhanany E, Laskar O, Schuster O, Rapid, sensitive and reliable ricin identification in serum samples using LC-MS/MS, Toxins (Basel) 13 (2021). 10.3390/toxins13020079 PMC7911523 33499033

[14] Fredriksson SA, Artursson E, Bergstrom T, Ostin A, Nilsson C, Astot C, Identification of RIP-II toxins by affinity enrichment, enzymatic digestion and LC- MS, Anal. Chem. 87 (2015) 967–974. 25496503 10.1021/ac5032918

[15] Schieltz DM, McWilliams LG, Kuklenyik Z, Prezioso SM, Carter AJ, Williamson YM, McGrath SC, Morse SA, Barr JR, Quantification of ricin RCA and comparison of enzymatic activity in 18 Ricinus communis cultivars by isotope dilution mass spectrometry, Toxicon 95 (2015) 72–83. 25576235 10.1016/j.toxicon.2015.01.003 PMC5303535

[16] Hodge DR, Prentice KW, Ramage JG, Prezioso S, Gauthier C, Swanson T, Hastings R, Basavanna U, Datta S, Sharma SK, Garber EA, Staab A, Pettit D, Drumgoole R, Swaney E, Estacio PL, Elder IA, Kovacs G, Morse BS, Kellogg RB, Stanker L, Morse SA, Pillai SP, Comprehensive laboratory evaluation of a highly specific lateral flow assay for the presumptive identification of ricin in suspicious white powders and environmental samples, Biosecur. Bioterror. 11 (2013) 237–250. 24320219 10.1089/bsp.2013.0053

[17] Bioclone Inc, BcMag™ amine-terminated magnetic beads, Retrieved from bioclone. us: http://www.bioclone.us/amine-terminated-magnetic-beads-particle-resin-matrix.html.

[18] McSweeney PLH, Fox PF, Advanced Dairy Chemistry: Volume 3: Lactose, Water, Salts and Minor Constituents, Springer, New York, 2009.

[19] Berg JM, Tymoczko JL, Stryer L, Biochemistry, fifth ed., Freeman WH, New York, 2002.

[20] Zentz C, Frenoy JP, Bourrillon R, Binding of galactose and lactose to ricin. Equilibrium studies, Biochim. Biophys. Acta 536 (1978) 18–26. 708758 10.1016/0005-2795(78)90047-8

[21] Gilliland SE, Speck ML, Woodard JR Jr., Stimulation of lactic streptococci in milk by -galactosidase, Appl. Microbiol. 23 (1972) 21–25. 4621795 10.1128/am.23.1.21-25.1972 PMC380271

